# pH-Responsive Tumor-Targetable Theranostic Nanovectors Based on Core Crosslinked (CCL) Micelles with Fluorescence and Magnetic Resonance (MR) Dual Imaging Modalities and Drug Delivery Performance

**DOI:** 10.3390/polym8060226

**Published:** 2016-06-07

**Authors:** Sidan Tian, Guhuan Liu, Xiaorui Wang, Guoying Zhang, Jinming Hu

**Affiliations:** Chinese Academy of Sciences Key Laboratory of Soft Matter Chemistry, Department of Polymer Science and Engineering, University of Science and Technology of China, Hefei 230026, China; sdtian@mail.ustc.edu.cn (S.T.); ghliu@ustc.edu.cn (G.L.); wxr114@mail.ustc.edu.cn (X.W.); gyzhang@ustc.edu.cn (G.Z.)

**Keywords:** pH-responsive, tumor targeting, aggregation induced emission, MR imaging, pHLIP

## Abstract

The development of novel theranostic nanovectors is of particular interest in treating formidable diseases (e.g., cancers). Herein, we report a new tumor-targetable theranostic agent based on core crosslinked (CCL) micelles, possessing tumor targetable moieties and fluorescence and magnetic resonance (MR) dual imaging modalities. An azide-terminated diblock copolymer, *N*_3_-POEGMA-*b*-P(DPA-*co*-GMA), was synthesized via consecutive atom transfer radical polymerization (ATRP), where OEGMA, DPA, and GMA are oligo(ethylene glycol)methyl ether methacrylate, 2-(diisopropylamino)ethyl methacrylate, and glycidyl methacrylate, respectively. The resulting diblock copolymer was further functionalized with DOTA(*Gd*) (DOTA is 1,4,7,10-tetraazacyclododecane-1,4,7,10-tetrakisacetic acid) or benzaldehyde moieties via copper(I)-catalyzed alkyne-azide cycloaddition (CuAAC) chemistry, resulting in the formation of DOTA(*Gd*)-POEGMA-*b*-P(DPA-*co*-GMA) and benzaldehyde-POEGMA-*b*-P(DPA-*co*-GMA) copolymers. The resultant block copolymers co-assembled into mixed micelles at neutral pH in the presence of tetrakis[4-(2-mercaptoethoxy)phenyl]ethylene (TPE-4SH), which underwent spontaneous crosslinking reactions with GMA residues embedded within the micellar cores, simultaneously switching on TPE fluorescence due to the restriction of intramolecular rotation. Moreover, camptothecin (CPT) was encapsulated into the crosslinked cores at neutral pH, and tumor-targeting pH low insertion peptide (pHLIP, sequence: AEQNPIYWARYADWLFTTPLLLLDLALLVDADEGTCG) moieties were attached to the coronas through the Schiff base chemistry, yielding a theranostic nanovector with fluorescence and MR dual imaging modalities and tumor-targeting capability. The nanovectors can be efficiently taken up by A549 cells, as monitored by TPE fluorescence. After internalization, intracellular acidic pH triggered the release of loaded CPT, killing cancer cells in a selective manner. On the other hand, the nanovectors labeled with DOTA(*Gd*) contrast agents exhibited increased relaxivity (*r*_1_ = 16.97 mM^−1^·s^−1^) compared to alkynyl-DOTA(*Gd*) small molecule precursor (*r*_1_ = 3.16 mM^−1^·s^−1^). Moreover, *in vivo* MRI (magnetic resonance imaging) measurements revealed CCL micelles with pHLIP peptides exhibiting better tumor accumulation and MR imaging performance as well.

## 1. Introduction

The combination integration of diagnostic and therapeutic functionalities into one system generates theranostic platforms, which is quite appealing because of the potential for developing personalized nanomedicine [1,2,3,4]. In this context, tremendous research has been carried out on the basis of inorganic nanoparticles [5], supramolecular assemblies [6,7], and hybrid materials [8,9], which are integrated with diverse imaging modalities and therapeutic agents. Of these, the incorporation of fluorescence [10,11] and magnetic resonance (MR) imaging [12,13] modalities represents a popular design strategy by taking advantage of high sensitivity and resolution of the former, as well as the invasiveness and high tissue penetration of the latter. On the other hand, compared to inorganic nanoparticle-based nanovectors, supramolecular assemblies stand out due to facile accessibility, scalability, and multifunctional integration [7,14,15,16]. Therefore, it is of interest to develop supramolecular assembly-based theranostic nanovectors with fluorescence and MR dual imaging modalities.

Although it is quite convenient to incorporate fluorophores into supramolecular systems via either covalent or noncovalent approaches [17,18,19], there are still several concerns that need to be resolved. For example, if the fluorophores are noncovalently encapsulated, inevitable diffusion of the fluorophores from the nanocarriers into the surrounding environments renders the exact concentrations of loaded dyes imprecise. In contrast, the covalent linkage approach alleviates the diffusion issue, while it may also suffer from tedious synthetic protocols [20]. On the other hand, it is generally accepted that supramolecular assemblies are subjected to high dilution and vigorous shearing force after intravenous administration, likely deforming or even disintegrating the supramolecular assemblies [21,22,23], which may lead to premature drug release and thus systemic toxicity [24,25,26,27]. Therefore, it is necessary to fix the microstructure of assemblies before arriving at the sites of action. To this end, a number of strategies have been applied such as shell/core crosslinking (SCL/CCL) and unimolecular assemblies to maintain the assembly microstructures during blood circulation [28,29,30,31,32,33,34,35,36,37,38,39,40,41].

Note that the crosslinking approaches reinforce the microstructures of the supramolecular assemblies, and thus, in return, impede the movements of the polymeric chains. Given that the fluorescence of chromophores with aggregation-induced emission (AIE) characteristics could be selectively switched on if the intramolecular rotation is restricted [42,43,44,45,46,47,48], we thus hypothesized that it could be possible to fix the assembly microstructure and synchronously introduce fluorescence imaging modality into the assembly system by taking advantage of AIE chromophore-mediated crosslinking reaction.

**Scheme 1:**
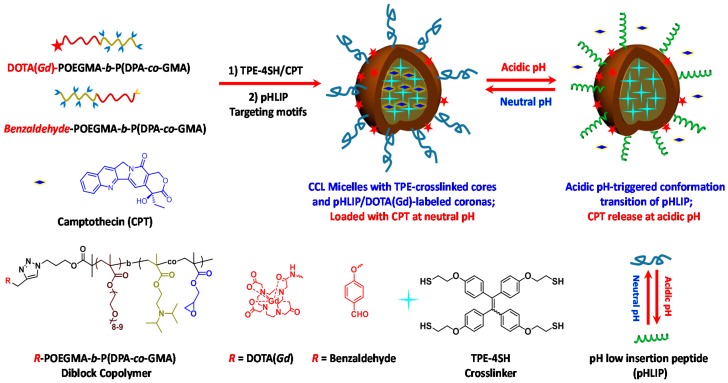
Schematic illustration of the fabrication of the CCL (core cross-linked) micelles with fluorescence and magnetic resonance (MR) dual imaging modalities from DOTA(*Gd*)-POEGMA-*b*-P(DPA-*co*-GMA) and benzaldehyde-POEGMA-*b*-P(DPA-*co*-GMA) diblock copolymers and tetrakis[4-(2-mercaptoethoxy)phenyl]ethylene (TPE-4SH) fluorophores via co-assembly and the spontaneous thiol-epoxy click chemistry. The CCL micelles were further functionalized with pH-low insertion peptide (pHLIP) through the formation of Schiff base linkages, followed by reduction with sodium cyanoborohydride (NaBH_3_CN). Under neutral pH condition, the pHLIP was in a coil state while it was transformed to α-helical conformation under mildly acidic pH, leading to increased cellular internationalization and allowing for fluorescence and MR imaging in live cells. Simultaneously, pH-triggered protonation of DPA moieties led to the release of encapsulated camptothecin (CPT) drug. Red stars: DOTA(*Gd*) moieties; arrows: TPE fluorophore; diamond: CPT drug.

To verify our hypothesis, herein, a block copolymer of *N*_3_-POEGMA-*b*-P(DPA-*co*-GMA), was firstly synthesized via successive atom transfer radical polymerization (ATRP) using an azide-terminated initiator, 3-azidopropyl 2-bromo-2-methylpropanoate, where OEGMA, DPA, and GMA are oligo(ethylene glycol)methyl ether methacrylate, 2-(diisopropylamino)ethyl methacrylate, and glycidyl methacrylate, respectively. The resulting diblock copolymer was further functionalized with T_1_-type MR imaging contrast agent, alkynyl-DOTA(*Gd*) [12,49], and 4-(prop-2-ynyloxy)benzaldehyde through copper(I)-mediated alkyne-azide cycloaddition (CuAAC) click reactions, affording DOTA(*Gd*)-POEGMA-*b*-P(DPA-*co*-GMA) and benzaldehyde-POEGMA-*b*-P(DPA-*co*-GMA). The as-prepared diblock copolymers co-assembled into mixed micellar nanoparticles under neutral pH milieu, with DOTA(*Gd*) and benzaldehyde functionalities at the peripheral of the nanoparticles [50,51,52,53,54,55]. Given the presence of GMA moieties within the micellar cores, tetrakis[4-(2-mercaptoethoxy)phenyl]ethylene (TPE-4SH) with four thiol groups, a well-documented chromophore with unique AIE properties [56], was encapsulated into the micellar cores during the co-assembly process, and spontaneous thiol-epoxy click reaction within the cores then occurred [57,58], crosslinking the micellar nanoparticles and switching on the fluorescence of TPE moieties. To confer the as-assembled micelles with selective targeting performance toward tumor tissues, pH low insertion peptide (pHLIP) was incorporated onto the micellar surfaces by means of Schiff base chemistry with the formation of imine bonds between benzaldehyde and pHLIP [59]. pHLIP, well investigated in a number of innovative studies by Engleman and coworkers, was known as one kind of pH-dependent membrane insertion peptide and formed an α-helical structure under mildly acidic pH conditions [60,61,62,63], remarkably boosting the internalization of as-labeled entities [64,65,66,67,68,69,70,71]. Under neutral pH, the CCL micellar nanoparticles could be employed to load hydrophobic drug (e.g., camptothecin, CPT), thus generating a tumor-targetable CCL micelle-based theranostic nanovector with fluorescence and MR dual imaging performance. The cellular internalization of the nanovector could be monitored by the TPE fluorescence and intracellular release of CPT could be actuated by mildly acidic pH due to the swelling of the micellar cores resulting from the protonation of DPA moieties [72,73,74,75,76,77,78,79,80,81]. Furthermore, after intravenous administration, the selective accumulation in tumor tissues of CCL micellar nanoparticles, by taking advantage of targeting pHLIP, was visualized by MR imaging (Scheme 1). The combination of AIE chromophores and CCL technique provides a convenient way to fabricate theranostic agents with stable microstructure and fluorescence imaging capability, while the multifunctional integration nature of supramolecular assemblies further extend the design scope of new theranostic nanovectors and may pave the way for follow-up clinical assessments.

## 2. Materials and Methods

### 2.1. Materials

Oligo(ethylene glycol) monomethyl ether methacrylate (OEGMA, *M*_n_ = 475 g/mol, mean degree of polymerization, DP, is 8–9) was purchased from Sigma-Aldrich Corporation (Shanghai, China) and was passed through a neutral alumina column to remove the inhibitor and then stored at −20 °C prior to use. Glycidyl methacrylate (GMA) was purchased from Sinopharm Chemical Reagent Co., Ltd. (Shanghai, China) and purified by vacuum distillation prior to use. 2-(Diisopropylamino)ethyl methacrylate (DPA) was purchased from Aldrich and purified by vacuum distillation prior to use. *N*,*N*,*N*′,*N*′′,*N*′′-Pentamethyldiethylenetriamine (PMDETA, 98%) and copper(I) bromide (CuBr, 98%) were purchased from Aldrich and used as received. Isopropanol, tetrahydrofuran (THF), *N*,*N*′-dimethylformamide (DMF), and all other reagents were purchased from Sinopharm Chemical Reagent Co., Ltd. (Hefei, China) and used as received. pHLIP (98%) was purchased from Chinapeptides Co., Ltd. (Shanghai, China) and used as received. Water was deionized with a Milli-Q SP reagent water system (Millipore, Billerica, MA, USA) to a specific resistivity of 18.4 MΩ·cm. 1,1,2,2-Tetrakis[4-(2-mercaptoethoxy)phenyl]ethane (TPE-4SH) [56], 3-azidopropyl 2-bromo-2-methylpropanoate [82], 4-(prop-2-ynyloxy)benzaldehyde [82], and alkynyl-functionalized DOTA(*Gd*) complex [83,84], alkynyl-DOTA(*Gd*) (DOTA is 1,4,7,10-tetraazacyclododecane-1,4,7,10-tetrakisacetic acid), were synthesized according to literature procedures.

### 2.2. Sample Preparation

**Scheme 2:**
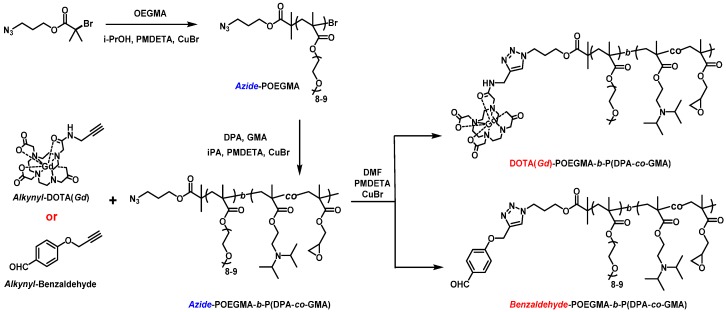
Synthetic routes of the preparation of MRI (magnetic resonance imaging) contrast agent-labeled DOTA(*Gd*)-POEGMA-*b*-P(DPA-*co*-GMA) and benzaldehyde-terminated benzaldehyde-POEGMA-*b*-P(DPA-*co*-GMA) diblock copolymers via consecutive atom radical transfer polymerizations (ATRP), followed by copper(I)-catalyzed alkyne-azide cycloaddition (CuAAC) reactions.

Synthetic routes employed for the preparation of amphiphilic block copolymers, POEGMA-*b*-P(DPA-*co*-GMA), are shown in Scheme 2.

#### 2.2.1. Synthesis of *N*_3_-POEGMA-Br Macroinitiator (Scheme 2)

3-Azidopropyl 2-bromo-2-methylpropanoate (0.250 g, 1.00 mmo1), OEGMA (47.50 g, 100.0 mmol), PMDETA (0.173 g, 1.00 mmol), and isopropanol (50 mL) were charged into a reaction flask. The mixture was degassed by three freeze-pump-thaw cycles and backfilled with nitrogen. After heating to 35 °C, CuBr (0.143 g, 1.00 mmol) was introduced as a solid under the protection of N_2_ to start the polymerization. After stirring for 3 h, the reaction tube was quenched into liquid N_2_, opened and exposed to air, and diluted with 60 mL THF. Then, the mixture solution was passed through a silica gel column using THF as the eluent to remove copper catalysts. After removing all the solvents on a rotary evaporator, the residues were re-dissolved in THF and precipitated into an excess of cold *n*-hexane. The above dissolution–precipitation cycle was repeated three times. The final product was dried in a vacuum oven overnight at room temperature, yielding colorless viscous liquid (12.7 g, yield: 26.7%). The number average molecular weight (*M*_n_) and molecular weight distribution (*M*_w_/*M*_n_) of *N*_3_-POEGMA-Br were determined by GPC (gel permeation chromatography) using THF as the eluent, revealing an *M*_n_ of 17.1 kDa and *M*_w_/*M*_n_ of 1.09 (Table 1 and Figure S1a). To determine the degree of polymerization (DP) of POEGMA block, the resulting *N*_3_-POEGMA-Br homopolymer was treated with an excess amount of 4-(prop-2-ynyloxy)benzaldehyde (10 equivalent relative to the azide terminal) and the successful transformation of the azide terminal was evidenced by FT-IR spectra (Figure S2). Using the characteristic peaks of aromatic rings, the DP of benzaldehyde-POEGMA-Br macroinitiator was determined to be 32 by ^1^H NMR analysis (Figure S3A), and thus the aizde-containing precursor was denoted as *N*_3_-POEGMA_32_-Br (**P1**, Table 1).

#### 2.2.2. Synthesis of *N*_3_-POEGMA-*b*-P(DPA-*co*-GMA) Block Copolymers (Scheme 2)

*N*_3_-POEGMA-*b*-P(DPA-*co*-GMA) diblock copolymer was synthesized via the ATRP of DPA and GMA co-monomers using **P1** as the macroinitiator. Briefly, **P1** (1.50 g, 0.1 mmol), DPA (0.85 g, 4 mmol), GMA (0.14 g, 1 mmol), PMDETA (0.017 g, 0.10 mmol), and isopropanol (3 mL) were charged into a reaction flask. The mixture was degassed by three freeze-pump-thaw cycles and backfilled with nitrogen. After heating to 35 °C, CuBr (0.014 g, 0.10 mmol) was introduced as a solid under the protection of N_2_ to start the polymerization. After stirring for 6 h, the reaction tube was quenched into liquid N_2_, opened and exposed to air, and diluted with 30 mL THF. The mixture was then passed through a silica gel column using THF as the eluent to remove copper catalysts. After removing all the solvents on a rotary evaporator, the residues were dissolved in THF and precipitated into an excess of cold *n*-hexane. The above dissolution-precipitation cycle was repeated three times. The final product was dried in a vacuum oven overnight at room temperature, yielding a white solid (2.17 g, yield: 67.8%). The *M*_n_ and *M*_w_/*M*_n_ of *N*_3_-POEGMA_33_-*b*-P(DPA-*co*-GMA) were determined by GPC using THF as the eluent, revealing an *M*_n_ of 21.0 kDa and *M*_w_/*M*_n_ of 1.14 (Table 1 and Figure S1b). The conversions of OEGMA and GMA were determined to be 90% and 60%, respectively, based on ^1^H NMR analysis (Figure S3B) of the product after click reaction with 4-(prop-2-ynyloxy)benzaldehyde (Figure S4b). Thus, the as-synthesized diblock copolymer was denoted as *N*_3_-POEGMA_32_-*b*-P(DPA_0.86_-*co*-GMA_0.14_)_42_ (**BP1**, Table 1).

#### 2.2.3. Synthesis of DOTA(*Gd*)-POEGMA-*b*-P(DPA-*co*-GMA) and Benzaldehyde-POEGMA-*b*-P(DPA-*co*-GMA) Block Copolymers (Scheme 2)

**BP2** and **BP3** were obtained by the click reactions of **BP1** precursor with alkynyl-DOTA(*Gd*) and 4-(prop-2-ynyloxy)benzaldehyde, respectively. Using the preparation of **BP3** as an example, typically, **BP1** (2.3 g, 0.10 mmol azide moieties), 4-(prop-2-ynyloxy)benzaldehyde (0.16 g, 1.00 mmol), PMDETA (17 mg, 0.10 mmol), and DMF (5 mL) were charged into a glass ampoule equipped with a magnetic stirring bar. The mixture was degassed by three freeze-pump-thaw cycles, and then CuBr (14 mg, 0.10 mmol) was introduced under N_2_ protection. After thermostating at 60 °C in an oil bath and stirring for 12 h, the reaction tube was quenched into liquid N_2_, opened and exposed to air, and diluted with 30 mL THF. The reaction mixture was then passed through a basic alumina column using THF as the eluent to remove copper catalysts. After removal of all the solvents on a rotary evaporator, the residues were dissolved in THF and precipitated into an excess of cold *n*-hexane. The obtained white solid was further purified by dialysis (cellulose membrane, molecular weight cutoff: 3500 Da) against deionized water for 12 h, and then lyophilized as a white solid (2.01 g, yield: 87.3%). **BP3** with MR imaging contrast agent moieties, DOTA(*Gd*), at the chain terminal was also synthesized according to a similar protocol. The Gd^3+^ content within the block copolymer was determined to be 0.63% by inductively coupled plasma atomic emission spectrometry (ICP-AES) measurement.

#### 2.2.4. Fabrication of Core Crosslinked (CCL) Mixed Micelles of **BP2** and **BP3** Using Tetrakis[4-(2-mercaptoethoxy)phenyl]ethylene (TPE-4SH) as the Crosslinker

Mixed CCL micelles were co-assembled from **BP2**, **BP3**, and TPE-4SH via a co-solvent approach. Briefly, **BP2** (9 mg) and **BP3** (1 mg) and TPE-4SH (0.36 mg, 0.25 equivalent to the GMA residues) were dissolved in DMF (1 mL) with the total polymer concentration being 10.0 g/L. For the CPT loading micelles, CPT (1 mg) was added to the above solution. The DMF solution was then injected into deionized water (9 mL) in one-shot under continuously stirring for 48 h to allow the complete core crosslinking reaction. After that, the as-prepared CCL micelles were exhaustively dialyzed against deionized water and fresh deionized water was replaced approximately every 6 h. Finally, the micellar dispersion was diluted to desired concentrations for further experiments. Using a similar protocol, hydrophobic anticancer drug, camptothecin (CPT) was loaded into the CCL micellar cores and the CPT loading efficiency (DLE) was determined to be 6.4 wt % by UV–Vis absorption measurements.

#### 2.2.5. Functionalization of Mixed Micelles with Targeting Peptide

Typically, pHLIP (0.16 mg, 1 equivalent relative to the benzaldehyde residues) was added to the micellar solution and stirred for 2 h. After that, NaCNBH_3_ (0.0036 mg, 1.5 equivalent relative to the imine functionality) was then added to the solution to reduce the imine bonds with the formation of secondary amines. After stirring at room temperature for 1 h, excess small molecules and unreacted targeting peptide were then removed by dialysis against deionized water.

#### 2.2.6. *In Vitro* Cytotoxicity Assay

A549 cells were employed for *in vitro* cytotoxicity evaluation via MTT (3-(4,5-dimethyl-2-thiazolyl)-2,5-diphenyl-2-*H*-tetrazolium bromide) assay. A549 cells were first cultured in Dulbecco’s modified Eagle medium (DMEM) supplemented with 10% fetal bovine serum (FBS), penicillin (100 units/mL), and streptomycin (100 μg/mL) at 37 °C in a CO_2_/air (5:95) incubator for 2 days. For cytotoxicity assay, A549 cells were seeded in a 96-well plate at an initial density of ca. 5000 cells/well in 100 μL of complete DMEM medium. After incubating for 24 h, DMEM was replaced with fresh medium, and the cells were treated with polymer micellar solution at varying concentrations. The treated cells were incubated in a humidified environment with 5% CO_2_ at 37 °C for 48 h. The MTT reagent (in 20 μL PBS (phosphate buffer saline), 5 mg/mL) was added to each well. The cells were further incubated for 4 h at 37 °C. The medium in each well was then removed and replaced by 150 μL DMSO. The plate was gently agitated for 15 min before the absorbance at 570 nm was recorded by a microplate reader (Thermo Fisher, Waltham, MA, USA). Each experiment condition was done in quadruple, and the data are shown as the mean value plus a standard deviation (± SD).

#### 2.2.7. *In Vitro* MRI (Magnetic Resonance Imaging) Relaxivity Measurement

The longitudinal relaxation rates (1/*T*_1_) of pHLIP-functionalized mixed micelles of **BP2** and **BP3** (9/1, wt/wt) and small molecule contrast agent, alkynyl-DOTA(*Gd*), in aqueous solution at varying Gd^3+^ concentrations (0, 0.005, 0.01, 0.02, 0.03, and 0.04 mM) were acquired at room temperature using a GE Signa Horizon 1.5 T MR scanner (General Electric Company, Fairfield, CT, USA) equipped with a human shoulder coil. Conventional spin-echo pulse sequence was used for *T*_1_ measurements with a single slice thickness of 4 mm, field of view (FOV) of 10 × 10 cm, and matrix size of 128 × 128 pixels. The repetition times (TR) were 300, 400, 500, 600, 800, 1000, 2000, and 3000 ms with an echo time (TE) of 9 ms. The net magnetizations for each sample were determined from the selected region of interest (ROI) and fit to the following multiparametric nonlinear regression function: *M*_TR_ = *M*_0_(1 − e^−TR/*T*1^), where *M*_TR_ denotes the measured signal intensity as a functional of repetition time (TR) and *M*_0_ is the signal intensity in the thermal equilibrium. *T*_1_ were calculated from these data using a MATLAB program and longitudinal relaxivity *r*_1_ was determined from the slope of 1/*T*_1_ *versus* (Gd^3^^+^) plot.

#### 2.2.8. *In Vivo* MRI Measurement

Tumor-bearing BALB/c nude mice obtained with the following procedures. BALB/c nude mice were kept in a light- and temperature-controlled aseptic environment. A549 cells, which had been grown in DMEM plus 10% FBS, were collected and resuspended in PBS (pH 7.4). Approximately, 8–10 × 10^6^ cells were injected subcutaneously into the hind flank region of the BALB/c nude mice, and the tumors were allowed to grow. When the tumor volumes reached about 100 mm^3^, injections of CCL micelles with or without targeting pHLIP were performed at a dose of 0.1 μmol Gd^3+^/g, and 30 min later, the mice were taken for MR imaging (1.5 T, TR/TE = 800/13 ms; 25 °C).

### 2.3. Characterization

All nuclear magnetic resonance (^1^H NMR) spectra were recorded using CDCl_3_ as the solvent on a Bruker AV300 NMR (300 MHz for ^1^H) spectrometer (Bruker, Billerica, MA, USA) operated in the Fourier transform mode. Molecular weights and molecular weight distributions were determined by gel permeation chromatography (GPC) equipped with a Waters 1515 pump and Waters 2414 differential refractive index detector (Waters, Milford, MA, USA) (set at 30 °C). It used a series of two linear Styragel columns (HR2 and HR4) at an oven temperature of 45 °C. The eluent was THF at a flow rate of 1.0 mL/min. A series of low polydispersity polystyrene standards were employed for calibration. Fourier transform infrared spectroscopy (FT-IR) were recorded on a Bruker VECTOR-22 IR spectrometer (Bruker, Billerica, MA, USA). The spectra were collected over 64 scans with a spectral resolution of 4 cm^−1^. Inductively coupled plasma atomic emission spectrometry (ICP-AES) (Perkin Elmer Corporation Optima 7300 DV, Waltham, MA, USA) was used for Gd^3+^ content analysis. Dynamic laser light scattering (LLS) measurements were conducted on a Zeta nanosizer ZS (Malvern, Worcestershire, UK). All data were averaged over three consecutive measurements. All samples were filtered through 0.45 μm Millipore Acrodisc-12 filters (Billerica, MA, USA) to remove dust. Transmission electron microscopy (TEM) observations were conducted on a JEOL 2010 HRTEM (JEOL, Tokyo, Japan). The sample for TEM observations was prepared by placing 10 μL of micellar solution (0.5 g/L) on copper grids coated with thin films of Formvar. Confocal fluorescence images were taken on Leica SP2 (Leica, Wetzlar, Germany), using the 405 nm laser as the excitation light source.

## 3. Results and Discussion

As shown in Scheme 2, a *N*_3_-POEGMA-Br macroinitiator comprised of an azide functionality at the chain terminal was firstly synthesized via ATRP of OEGMA monomer using a 3-azidopropyl 2-bromo-2-methylpropanoate initiator. The successful living polymerization was evidenced by GPC elution profile (Figure S1a), revealing an *M*_n_ of 17.1 kDa and an *M*_w_/*M*_n_ of 1.09 (Table 1). Interestingly, the ATRP condition had no appreciable adverse effects on the stability of azide moieties, and the presence of azide terminal in *N*_3_-POEGMA-Br macroinitiator was confirmed by FT-IR spectrum (Figure S2a). To work out the exact degree of polymerization, DP, of the POEGMA block, an aromatic ring-containing 4-(prop-2-ynyloxy)benzaldehyde was conjugated onto the chain terminal through copper(I)-catalyzed alkyne-azide cycloaddition (CuAAC) reaction. The successful introduction of benzaldehyde derivatives was confirmed by the disappearance of azide signal in the FT-IR spectrum after click reaction (Figure S2b), and the simultaneous appearance of the characteristic resonance peaks of benzene rings in the ^1^H NMR spectrum (Figure S3A). Using the benzene ring signals as the reference standard, the DP of POEGMA block was determined to be 32 (Figure S3A). Thus, the resulting macroinitiator was denoted as *N*_3_-POEGMA_32_-Br (**P1**, Table 1).

Subsequently, chain extension with DPA and GMA co-monomers were carried out using **P1** macroinitiator. The as-prepared *N*_3_-POEGMA_32_-*b*-P(DPA-*co*-GMA) diblock copolymer exhibited an *M*_n_ of 21.0 kDa and an *M*_w_/*M*_n_ of 1.14 (Figure S1b, Table 1). Again, the azide-terminal was retained after copolymerization, as confirmed by the FT-IR spectrum (Figure S4a). Next, the azide groups on the diblock copolymer were functionalized with alkynyl-DOTA(*Gd*) contrast agent and 4-(prop-2-ynyloxy)benzaldehyde, affording DOTA(*Gd*)-POEGMA-*b*-P(DPA-*co*-GMA) and benzaldehyde-POEGMA-*b*-P(DPA-*co*-GMA), respectively. The successful post-modification with DOTA(*Gd*) contrast agents and benzaldehyde derivatives through high-fidelity click chemistry was corroborated by the disappearance of the azide moieties (Figure S4b,c). Moreover, the successful attachment of benzaldehyde derivatives at the chain terminal allowed for the calculation of the molar compositions of DPA and GMA monomers (Figure S3B), and the target diblock copolymer was denoted as benzaldehyde-POEGMA_32_-*b*-P(DPA_0.86_-*co*-GMA_0.14_)_42_ (**BP3**, Table 1).

In the next step, we investigated the self-assembly behavior of the resultant diblock copolymers. **BP2** and **BP3** diblock copolymer (9/1, wt/wt) can co-assemble into micellar nanoparticles with a *D*_h_ (hydrodynamic diameter) of 27 nm (Figure S5a) in aqueous solution at neutral pH (7.4). The formed micellar nanoparticles labeled with DOTA(*Gd*) on the micellar coronas could thus be employed as a macromolecular contrast agent. However, given dual imaging modalities providing more accurate information and the poor stability of conventional aggregates undergoing unwanted disintegration during blood circulation, we hypothesized that it would be favorable and convenient if another imaging modality could be installed and the assembled nanostructures could be reinforced at the same time. To this end, TPE-4SH fluorophore was chosen to crosslink the micellar nanostructures by taking advantage of thiol-epoxy click chemistry. Notably, TPE chromophore was recognized by unique AIE characteristics, and the crosslinking process was expected to restrict the intramolecular rotation of TPE moieties. Therefore, the successful incorporation of TPE moieties into the micellar nanoparticles was distinguished by fluorescence turn-on.

**Figure 1:**
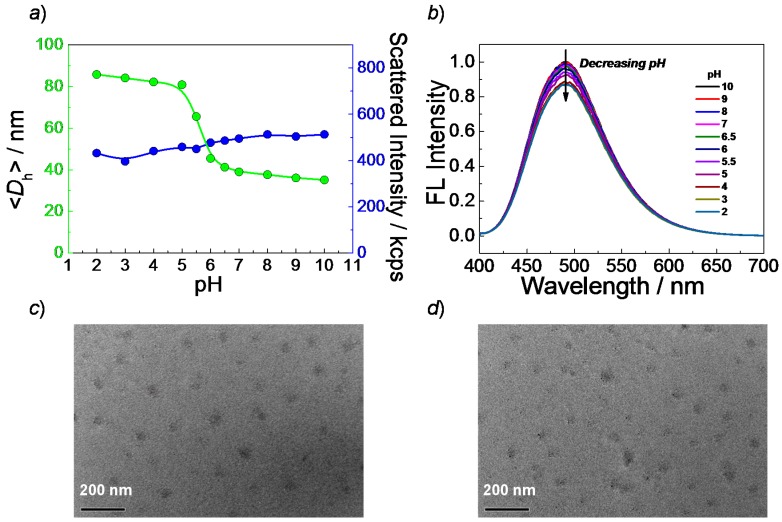
(**a**) Variations of hydrodynamic diameters, *D*_h_, and corresponding scattered light intensity changes obtained for 1.0 g/L aqueous solution (**BP2**/**BP3** = 9/1, wt %, 25 °C) of CCL(core cross-linked) micelles at varying pH values; (**b**) pH-dependence of normalized fluorescence emission spectra recorded for 1 g/L aqueous solution of the CCL micelles (25 °C, λ_ex_ = 390 nm; slit widths: Excitation 5 nm, Emission 5 nm) with pH decreasing from 10 to 2. TEM images of CCL micelles at (**c**) pH 10 and (**d**) pH 2.

To verify whether the thiol-epoxy click reaction can crosslink the mixed micellar nanoparticles, we examined the size changes of mixed micellar nanoparticles at varying pH values. Upon pH drop from 10 to 2, the hydrodynamic diameter, *D*_h_, of micellar nanoparticles increased from ~34 to ~87 nm, due to the swelling of the micellar cores as a result of the protonation of the DPA moieties at acidic pH conditions. Meanwhile, corresponding scattering intensity underwent a slight decrease within the same pH range, in accordance with the swelling of micellar nanoparticles. In sharp contrast, the mixed micelles without TPE-4SH addition underwent disintegration when the solution pH was decreased to 6.0 with a *D*_h_ of 7 nm (Figure S5a). Meanwhile, the scattering intensity experienced a significant drop in the absence of TPE-4SH crosslinker subjected to the same pH decrease (Figure S5b). Therefore, the DLS (dynamic laser scattering) result clearly suggested that, after the successful crosslinking of the mixed micellar cores, the micellar structures could be maintained instead of being disrupted even at acidic pH when DPA moieties were protonated (Figure 1a). Moreover, we observed significant fluorescence turn-on of TPE moieties after crosslinking reaction. The crosslinked micellar nanoparticles exhibited intense cyan fluorescence emission ascribed to TPE moieties, which underwent a steady decrease upon progressive pH drop from pH 10 to 2. The fluorescence intensity at pH 2 was 87% of that at pH 10, presumably due to the swelling of micellar cores, thus elevating intramolecular rotation of TPE moieties (Figure 1b). The evolution of fluorescence intensity further corroborated that the micellar nanoparticles was successfully crosslinked by means of thiol-epoxy reactions. Notably, the relatively stable fluorescence emission would be favorable for the intracellular imaging application in a wide pH range that were typically found in diverse organelles. Moreover, the successful crosslinking of micellar nanoparticles was also visualized by TEM observations, indicating the presence of micellar nanoparticles at both pH 10 and pH 2, regardless of protonation/deprotonation of DPA derivatives (Figure 1c,d).

**Figure 2:**
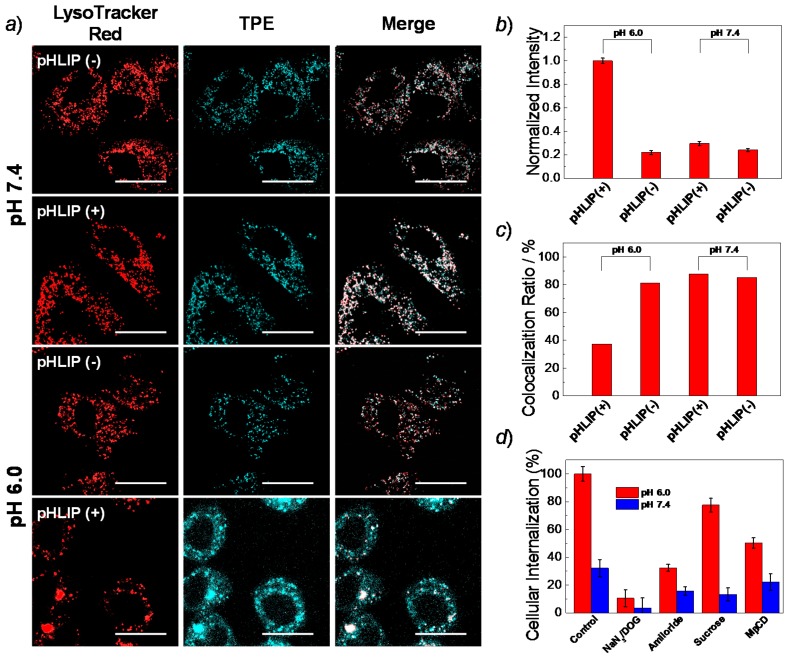
(**a**) Confocal laser scanning images (CLSMs) of A549 cells stained with LysoTracker Red and CCL micelles with or without pHLIP targeting peptide for 2 h at 37 °C. The final concentration of the CCL micelles in the culture medium was 0.05 g/L and the pH of the culture medium was adjusted to 6.0 with NH_4_Cl (scale bar: 50 μm; TPE (tetrakis[4-(2-mercaptoethoxy)phenyl]ethylene) channel: λ_ex_ = 405 nm, λ_em_ = 475 ± 25 nm; LysoTracker Red: λ_ex_ = 543 nm, λ_em_ = 605 ± 25 nm); (**b**) Normalized TPE fluorescence intensities within A549 cells quantified from CLSM observations; (**c**) Colocalization ratios of cyan fluorescence from CCL micelles and red emission of LysoTracker Red; and (**d**) Probing the mechanisms of cellular internalization using inhibitors of specific endocytic pathways at pH 7.4 and 6.0, respectively.

Next, we evaluated the cell internalization capability and examined the internalization pathways of CCL micelles by taking advantage of TPE fluorescence achieved by crosslinking micellar nanoparticles. Notably, although the fluorescence of CCL micelles slightly decreased at acidic pH (Figure 1b), it was bright enough to trace the CCL nanoparticles by confocal laser scanning microscopy (CLSM). To boost the intracellular uptake efficiency of CCL micellar nanoparticles, pHLIP targeting moieties were attached to the surfaces of micellar nanoparticles through the formation of imine bonds between targeting peptide and benzaldehyde residues. pHLIP can insert into lipid bilayers via the formation of *α*-helical structures under mildly acidic pH, thus enhancing the internalization of nanocarriers functionalized with pHLIP [64,65,66,67,68,70]. Although there were no significant changes in the fluorescence intensities of cyan channel for A539 cells after incubation either with the CCL micelles with targeting pHLIP at pH 7.4 or with CCL micelles without targeting pHLIP at both pH 7.4 and pH 6.0, pronounced fluorescence increase was observed for the A539 cells after incubation with CCL micelles with pHLIP at pH 6.0 (Figure 2a,b), suggesting an increased cellular accumulation of CCL nanoparticles. This result should be the contribution of targeting pHLIP on the micellar surfaces that form α-helical structures at pH 6.0, boosting the internalization of CCL nanoparticles. Additionally, quantitative colocalization analysis between the red channel of stained acidic organelles (e.g., endolysosomes) and the cyan channel of CCL nanoparticles revealed a lowest colocalization ratio (~40%) in the presence of targeting pHLIP at pH 6.0 (Figure 2b), while CCL micelles without pHLIP at both pH 7.4 and 6.0, and micelles with pHLIP at 7.4, exhibited similar high colocalization ratios (>80%). This result demonstrated that the internalized CCL micelles in the presence of targeting pHLIP at pH 7.4 and in the absence of targeting pHLIP at both pH 6.0 and 7.4 were primarily located within acidic organelles. However, CCL micelles with targeting pHLIP at acidic pH may circumvent the fate of nanoparticle-based delivery cargos that ends up in the endolysosomes, which was quite advantageous for the designing of efficient and robust nanocarriers.

The above specific cellular internalization performance of pHLIP-labeled CCL micellar nanoparticles at pH 6.0 encouraged us to further probe the internalization mechanism. Diverse endocytosis inhibitors were applied to evaluate the internalization pathway of the CCL micelles with pHLIP at pH 6.0. As shown in Figure 2d, NaN_3_/2-deoxyglucose (NaN_3_/DOG) was employed to inhibit the energy-dependent pathway [85], and the internalization of CCL micelles with targeting pHLIP at pH 6.0 was significantly suppressed subjected to NaN_3_/DOG treatment (~90% decrease compared to the control). Meanwhile, the addition of amiloride, an inhibitor of pinocytosis [86], resulted in a ~70% internalization decrease, indicating that pinocytosis also played an important role in the internalization of CCL micelles. By contrast, sucrose treatment and the introduction of methyl-β-cyclodextrin (MβCD) that blocked clathrin-mediated endocytosis and caveolae-mediated endocytosis [87,88], respectively, led to ~20% and ~45% internalization decrease, suggesting that these two pathways contributed less on the internalization of CCL micelles decorated with targeting pHLIP. This result concurred well with the intracellular distribution of pHLIP-labeled CCL micelles exhibiting the lowest colocalization ratio with LysoTracker red at pH 6.0 (Figure 2c). However, when A549 cells were incubated with CCL micelles at pH 7.4 in the presence of targeting pHLIP, the energy-dependent endocytosis pathway dominated the uptake process, while other internalization pathways were less affected by the presence of corresponding inhibitors, although cellular internalizations experienced holistic decreases with varying degrees compared to that at pH 6.0 (Figure 2d). This distinct performance on the cellular uptake was presumably ascribed to the pH-induced conformation transition of targeting pHLIP derivatives.

**Figure 3:**
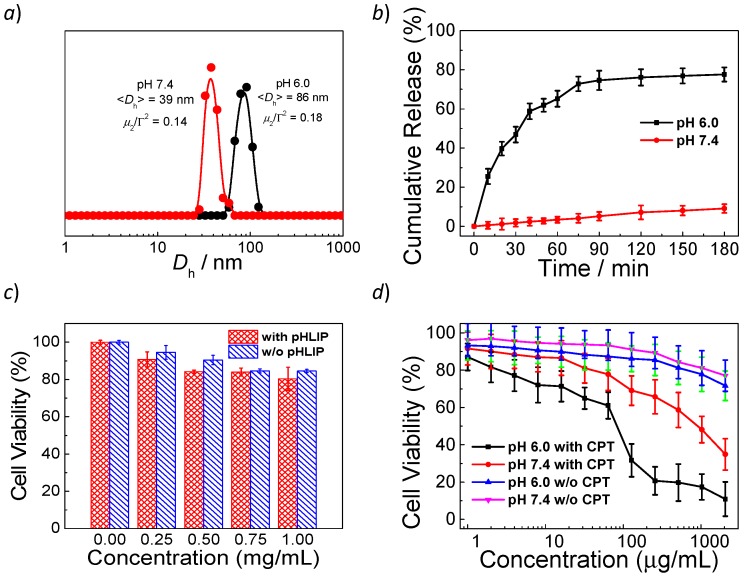
(**a**) Hydrodynamic diameter distributions of camptothecin (CPT)-loaded CCL micelles at pH 7.4 and pH 6.0, respectively; (**b**) CPT release profiles from CCL micelles at pH 7.4 and 6.0; (**c**) *In vitro* cytotoxicity of CCL micelles with and without pHLIP as determined by MTT (3-(4,5-dimethyl-2-thiazolyl)-2,5-diphenyl-2-*H*-tetrazolium bromide) assay against A549 cells at pH 7.4; and (**d**) *in vitro* cytotoxicity of pHLIP-decorated CCL micelles with or without CPT internalized under different pH as determined by MTT assay against A549 cells. For the cytotoxicity tests, A549 cells were firstly incubated with micelles for 2 h and were washed three time with PBS buffer, followed by a further incubation for 24 h.

After approval of the intracellular uptake capability of CCL micelles, we surmised that the pH-responsive CCL micelles with targeting moieties may be eligible for drug delivery application. The cellular internalization of the CCL micelles should be able to be monitored by fluorescence technique and the drug release could be regulated by intracellular pH by taking advantage of the pH-responsive nature of DPA moieties. Using chemotherapeutic CPT as an example, we found that CPT could be loaded into the micellar cores at pH 7.4 with a drug loading efficiency of 6.4 wt %. The CPT-loaded CCL micelles had a *D*_h_ of 39 nm at pH 7.4, which was similar to that of CPT-free micelles at pH 7.4 and increased to 86 nm at pH 6.0 due to the protonation of DPA moieties (Figure 3a). The CPT-release profiles were highly pH-dependent. For example, less than 10% drug release was achieved at pH 7.4 within 3 h, whereas over 80% drug was released within the same time range at pH 6.0. This drastic release performance was tentatively attributed to the fast protonation of DPA derivatives, followed by a speedy hydrophobicity-to-hydrophilicity transition, which was quite different from other drug nanocarriers implementing slow drug release profiles by means of other transitions with relatively slow kinetics [73,74,75,76]. Notably, *in vitro* cytotoxicity tests at pH 7.4 revealed that the targeting pHLIP derivatives and CCL micelles with a concentration up to 1.0 g/L without drug-loading were nontoxic to A549 cells (Figure 3c). However, upon incubation, A549 cells with the pHLIP-decorated CPT-loaded CCL micelles pronouncedly increased cytotoxicity was observed at both pH 7.4 and pH 6.0 (Figure 3d), although the cytotoxicity was lower than that of free CPT drug (Figure S6). For example, less than 10% cell viability was observed when the A549 cells were treated with 2.0 g/L CCL micelles loaded with CPT. Importantly, given CPT-loaded CCL micelles being more toxic at pH 6.0 than that at pH 7.4, we inferred that the targeting pHLIP on the micelle surfaces that enhanced cellular internalization of CCL micelles may lead to an increased CPT concentration in tumor cells, thereby killing tumor cells in a more efficient manner.

**Figure 4:**
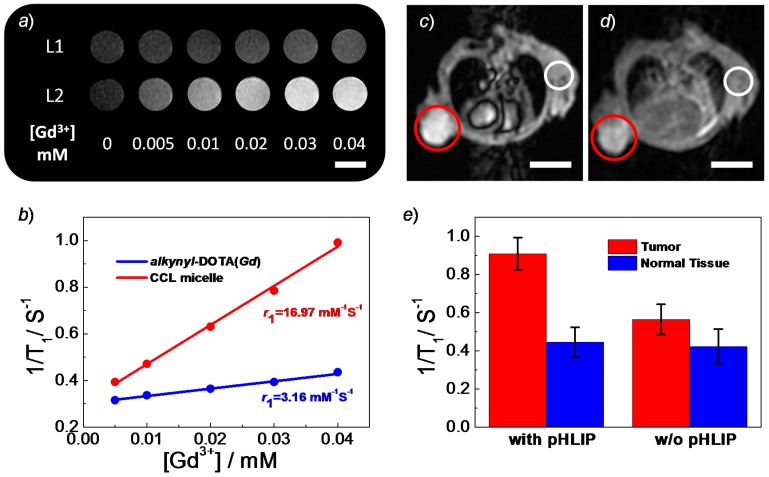
(**a**) *T*_1_-weighted spin-echo magnetic resonance (MR) imaging recorded for aqueous solutions of alkynyl-DOTA(*Gd*) (L1) and DOTA(*Gd*)-labeled CCL micelles (L2) with varying Gd^3+^ concentrations (0–0.04 mM); (**b**) water proton longitudinal relaxation rate (1/*T*_1_) of alkynyl-DOTA(*Gd*) small molecule and DOTA(*Gd*)-functionalized CCL micelles in aqueous solution as a function of Gd^3+^ concentration; (**c,d**) MR imaging of tumor-bearing BALB/c nude mice with 30 min incubation after intravenous injection of the aqueous solution of CCL micelles (**c**) with pHLIP and (**d**) without pHLIP (1.5 T, TR/TE = 800/13 ms; 25 °C). Injection was performed at a dosage of 0.1 μmol Gd^3+^/g. The xenograft tumors of A549 cells are circled in red and the normal tissues are labeled in white; and (**e**) quantitative analysis of 1/*T*_1_ as calculated from images (**c**) and (**d**). The scale bars in (**a**), (**c**), and (**d**) are 1 cm.

Building on the above results, we concluded that the pH-responsive CCL micelles with AIE characteristics and drug-loading capability could be employed as a fluorescence image-guided theranostic nanovector. Considering the presence of DOTA(*Gd*) contrast agents on the micelle surfaces, in the following work, we further appraised the MR imaging capability of the CCL micelles. Both DOTA(*Gd*) small molecules and mixed micelle-based macromolecular MRI contrast agent exhibited positive imaging contrast. The latter, however, possessed brighter contrast capability than for small molecular precursors at the same Gd^3+^ ion concentration, probably due to an increased local concentration and inhibited tumbling of DOTA(*Gd*) moieties [89]. Specifically, quantitative analysis revealed that the relaxivity (*r*_1_) of DOTA(*Gd*) small molecules and mixed micelle-based MRI contrast agents were of 3.16 and 16.97 mM^−1^·s^−1^, respectively. Note that the increased relaxivity was apparently advantageous for the *in vivo* imaging because of enhanced contrast. Tumor-bearing BALB/c nude mice with xenograft tumors of A549 cells were used for *in vivo* MR imaging, and the MR images were taken 30 min after intravenous injection of the aqueous solution of CCL micelles with or without pHLIP. It was revealed that pHLIP-labeled mixed micelles had a better imaging contrast than those without targeting peptides (Figure 4c,d), and the quantitative analysis of 1/T_1_ demonstrated that the CCL micelles with targeting peptides favorably accumulated within tumors instead of normal tissues (Figure 4e), as a result of the selectively targeting capability of pHLIP peptide under a mildly acidic circumstance typically observed in pathological regions (e.g., tumors). Taken together, the pH-responsive CCL micelles crosslinked with TPE moieties can not only intracellularly deliver therapeutic drugs with pH-dependent release profiles but also visualize the biodistribution and intracellular internalization process by means of MR and fluorescence imaging modalities.

## 4. Conclusions

In summary, a mixed micelle-based theranostic agent featuring fluorescence and MR dual modality imaging capability and pH-mediated drug release profile was fabricated. Block copolymers consisting of pendent epoxy residues within the pH-responsive blocks, POEGMA-*b*-P(DPA-*co*-GMA), was synthesized via ATRP technique, followed by introduction of MR imaging contrast agent and benzaldehyde moieties via CuAAC click chemistry. The as-prepared block copolymers self-assembled into micellar nanoparticles in aqueous solution at neutral pH, and crosslinking then occurred within the micellar cores in the presence of TPE-4SH, which was simultaneously loaded into the hydrophobic cores of the micellar nanoparticles during the self-assembly process. Notably, the crosslinking process of micellar nanoparticles was characterized by a drastic fluorescence turn-on of TPE moieties. The integration of AIE chromophores and highly efficient thiol-epoxy click chemistry enables the fabrication of theranostic nanovectors with long-term stability, simultaneously conferring the nanovectors with fluorescence imaging capability. Moreover, tumor-targetable pHLIP was attached to the peripheral of micellar nanoparticles via the formation of imine bonds, exhibiting a pH-dependent cellular internalization performance. Interestingly, after conjugation with pHLIP, the internalization pathway of CCL micelles was less dependent on clathrin-mediated endocytosis at pH 6.0 due to the pH-induced conformation transformation of pHLIP targeting entities. After encapsulation of therapeutic drug CPT, the drug release profiles of CPT were highly pH-dependent, and the CPT release was accelerated under acidic pH by means of pH-induced hydrophobic-to-hydrophilic transition of micelle cores. Moreover, the mixed micelle-based theranostic nanovector bearing MR imaging contrast agent in the coronas had a *r*_1_ of 16.97 mM^−1^s^−1^, an approximately 5.4-fold increase compared with the small molecular precursor. Furthermore, an *in vivo* MR imaging study revealed that the micelle-based macromolecular contrast agent possessed an enhanced accumulation in tumor tissues by taking advantage of the extracellular mildly acidic pH of tumor tissues that facilitated the conformation change of pHLIP targeting moieties. Further assessments on the theranostic performance of the CCL micelles are currently underway.

## Figures and Tables

**Table 1:** Structural parameters of block copolymers used in this study.

Entry	Samples ^a^	*M*_n_^a^ (kDa)	*M*_n_^b^ (kDa)	*M*_w_/*M*_n_^b^
**P1**	*N_3_*-POEGMA_32_-Br	17.6	17.1	1.09
**BP1**	*N_3_*-POEGMA_32_-*b*-P(DPA_0.86_-*co*-GMA_0.14_)_42_	26.1	21.0	1.14
**BP2**	DOTA(*Gd*)-POEGMA_32_-*b*-P(DPA_0.86_-*co*-GMA_0.14_)_42_	–	21.8	1.18
**BP3**	Benzaldehyde-POEGMA_32_-*b*-P(DPA_0.86_-*co*-GMA_0.14_)_42_	26.1	22.4	1.16

^a^ Determined by ^1^H NMR in D_2_O/DCl at pH 2; ^b^ The number average molecular weight (*M*_n_) and molecular weight distribution (*M*_w_/*M*_n_) of the copolymers were determined by GPC (gel permeation chromatography) using tetrahydrofuran (THF) as the eluent. OEGMA, DPA, and GMA are oligo(ethylene glycol)methyl ether methacrylate, 2-(diisopropylamino)ethyl methacrylate, and glycidyl methacrylate, respectively. DOTA is 1,4,7,10-tetraazacyclododecane-1,4,7,10-tetrakisacetic acid.

## Supplementary Materials

Supplementary Materials can be found at www.mdpi.com/2073-4360/8/6/226/s1.
