# Supplementary material for: pH-Responsive Tumor-Targetable Theranostic Nanovectors Based on Core Crosslinked (CCL) Micelles with Fluorescence and Magnetic Resonance (MR) Dual Imaging Modalities and Drug Delivery Performance
Source: Polymers (Basel). 2016 Jun 7;8(6):226. doi: 10.3390/polym8060226 (PMC6432225; doi:10.3390/polym8060226)
Supplement: Supplementary file 1 [file polymers-08-00226-s001.pdf]

Supplementary Materials: pH-Responsive Tumor-Targetable Theranostic Nanovectors Based on Core Crosslinked (CCL) Micelles with Fluorescence and Magnetic Resonance (MR) Dual Imaging Modalities and Drug Delivery Performance

Sidan Tian, Guhuan Liu, Xiaorui Wang, Guoying Zhang and Jinming Hu

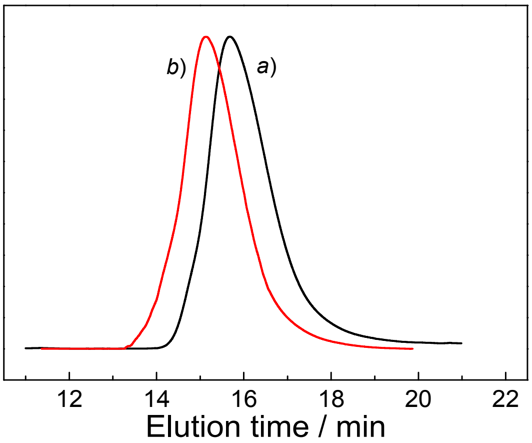

Figure S1. THF GPC traces of (**a**) N_3-POEGMA$_{32}$-Br macroinitiator and (**b**) N_3-POEGMA$_{32}$-*b*-P(DPA$_{0.86}$-*co*-GMA$_{0.14}$)$_{42}$ diblock copolymer.

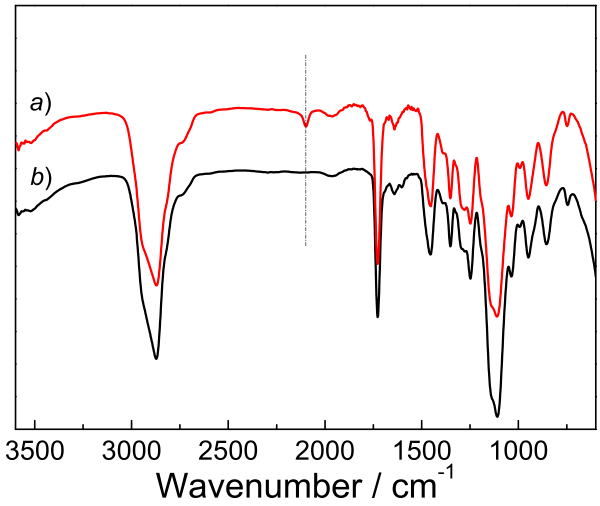

Figure S2. FT-IR spectra of N_3-POEGMA$_{32}$-Br macroinitiator (**a**) before and (**b**) after click reaction with 4-(prop-2-ynyloxy)benzaldehyde.

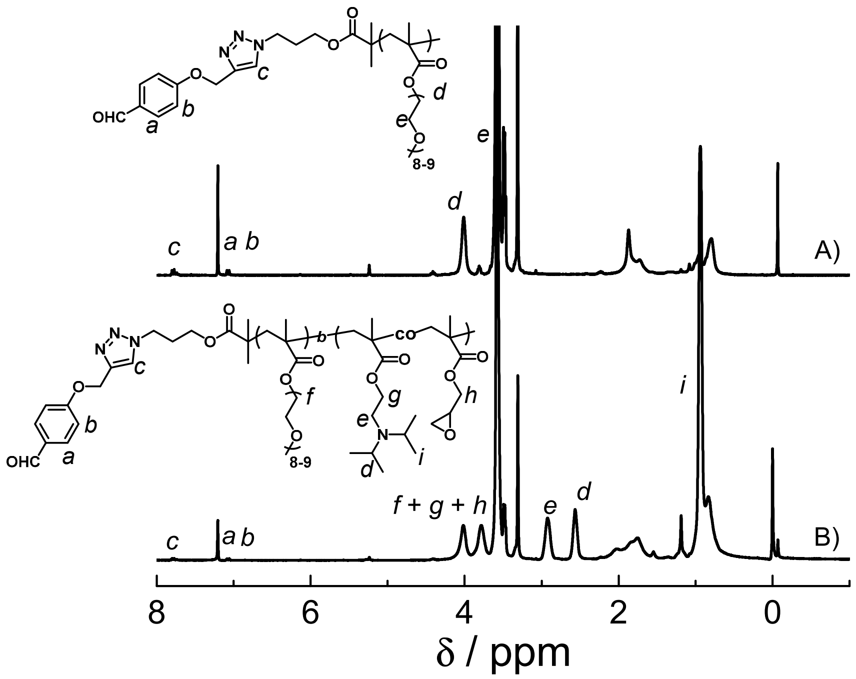

Figure S3. ^{1}H NMR spectra recorded in $CDCl_3$ for (**A**) benzaldehyde-POEGMA$_{32}$-Br and (**B**) benzaldehyde-POEGMA$_{32}$-*b*-P(DPA$_{0.86}$-*co*-GMA$_{0.14}$)$_{42}$ diblock copolymer.

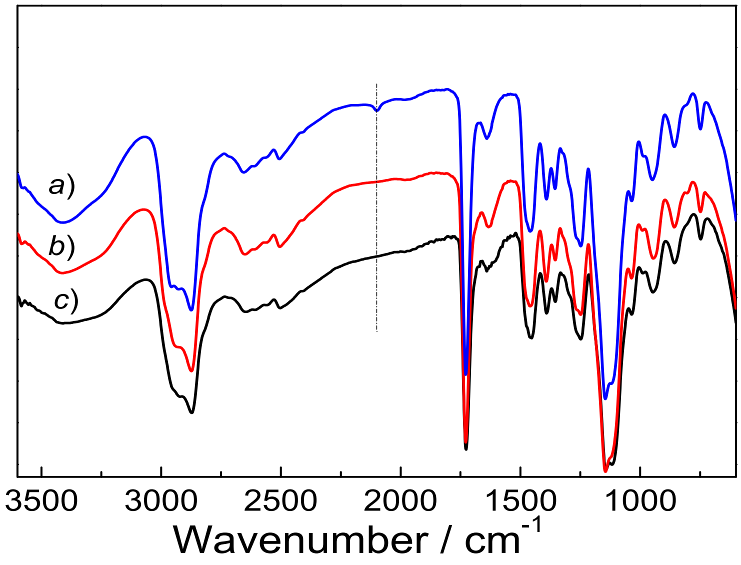

Figure S4. FT-IR spectra of (**a**) *N*$_3$-POEGMA$_{32}$-*b*-P(DPA$_{0.86}$-*co*-GMA$_{0.14}$)$_{42}$ (**BP1**) diblock copolymer precursor, (**b**) benzaldehyde-POEGMA$_{32}$-*b*-P(DPA$_{0.86}$-*co*-GMA$_{0.14}$)$_{42}$ (**BP3**), and (**c**) DOTA(*Gd*)-POEGMA$_{32}$-*b*-P(DPA$_{0.86}$-*co*-GMA$_{0.14}$)$_{42}$ (**BP2**).

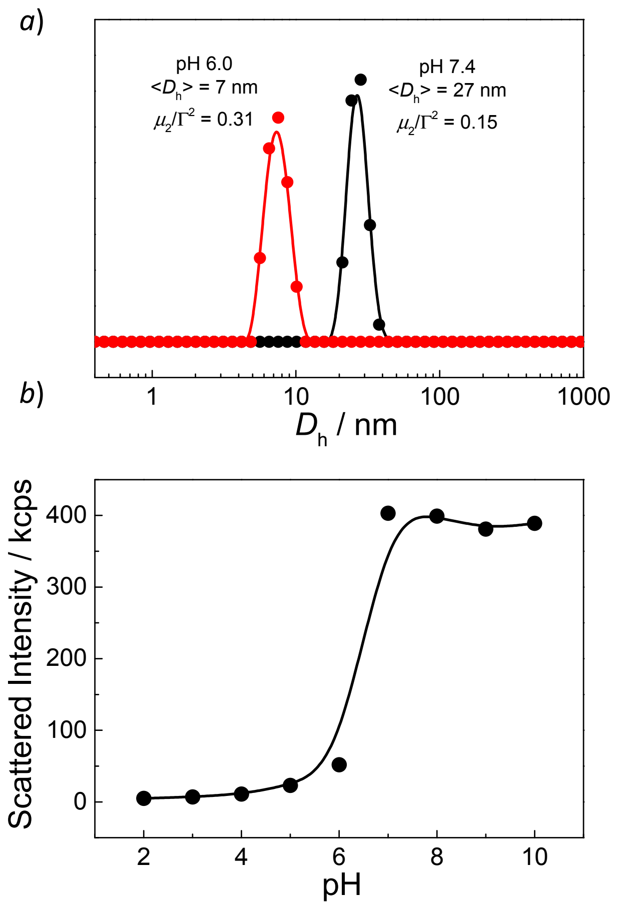

Figure S5. (**a**) Hydrodynamic distributions of 1.0 g/L aqueous solution (**BP2**/**BP3** = 9/1, wt %, 25 °C) of non-crosslinked *micelles* at pH 6.0 and 7.4; (**b**) Scattered light intensity changes for 1.0 g/L aqueous solution (**BP2**/**BP3** = 9/1, wt %, 25 °C) of non-crosslinked micelles at varying pH values.

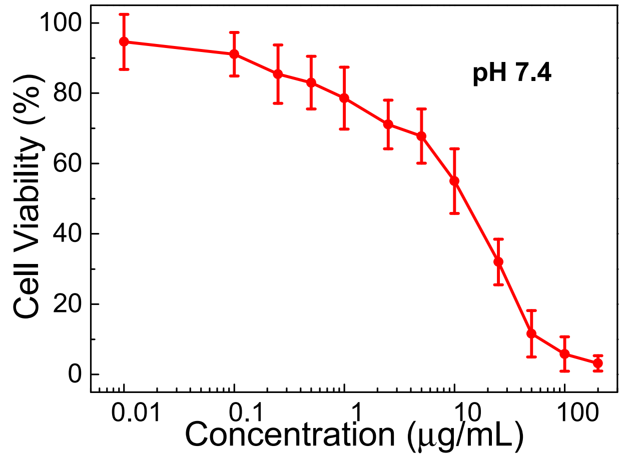

Figure S6. *In vitro* cytotoxicity of CPT at pH 7.4 as determined by MTT assay against A549 cells. For the cytotoxicity tests, A549 cells were firstly incubated with CPT at pH 7.4 for 2 h and were washed three time with PBS buffer, followed by a further incubation for 24 h.

© 2016 by the authors; licensee MDPI, Basel, Switzerland. This article is an open access article distributed under the terms and conditions of the Creative Commons Attribution (CC-BY) license (http://creativecommons.org/licenses/by/4.0/).
